# Electron interaction with laser-desorbed thymidine and guanine in the gas phase

**DOI:** 10.1140/epjd/s10053-025-01023-9

**Published:** 2025-06-30

**Authors:** Debasish Parida, Jiakuan Chen, Lara Schorr, Vy T. T. Nguyen, Muhammad Saqib, Andreas Bayer, Fabio Zappa, Stephan Denifl

**Affiliations:** 1 Institut für Ionenphysik und Angewandte Physik, Universität Innsbruck, Technikerstrasse 25, 6020 Innsbruck, Austria; 2 Center for Molecular Biosciences Innsbruck, Universität Innsbruck, Technikerstrasse 25, 6020 Innsbruck, Austria

## Abstract

**Abstract:**

In the present study we investigated electron attachment to the nucleoside thymidine (Td) and the nucleobase guanine (G) using a laser desorption source to transfer the compounds into the gas phase. Previous studies with Td indicated that the compound is thermally labile and may degrade upon thermal heating in standard molecular beam source. The present negative ion mass spectra for laser-desorbed Td and resistively heated Td share the same most three abundant fragment anions. Among those is the dehydrogenated parent anion (Td-H)^−^ which is strongly enhanced for laser-desorbed Td. We also find a considerable change of the fragmentation pattern for less abundant peaks in the mass spectra as well as changed characteristic in the total and mass selected anion efficiency curves of fragment anions. Electron attachment to G proceeds predominantly at electron energies below 3 eV. We ascribe this property to formation of a dipole-bound anion acting as a precursor state for efficient formation of the dehydrogenated anion (G-H)^−^. The present results complement previous electron attachment studies with other nucleobases showing that the dehydrogenated parent anion is the most abundant fragment anion for G as well.

**Graphic Abstract:**

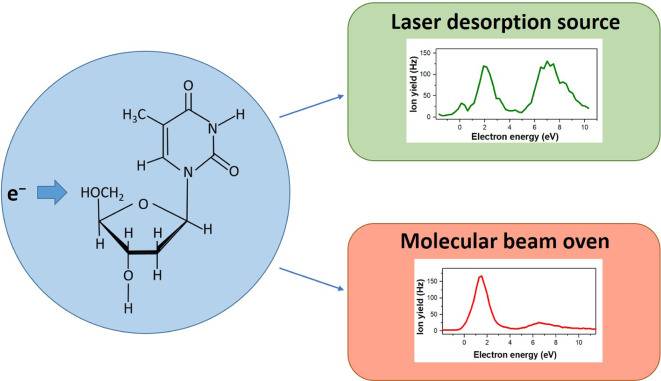

**Supplementary Information:**

The online version contains supplementary material available at 10.1140/epjd/s10053-025-01023-9.

## Introduction

At the beginning of this century, the possible potential of low-energy electrons in damaging DNA has been underlined by first pioneering experiments by Sanche and co-workers. They demonstrated that single and double strand breaks are induced in plasmid DNA after irradiation with electrons with an energy below 20 eV [1, 2]. Peaks in the yield of strand breaks as function of initial electron energy indicated dissociative electron attachment (DEA) as underlying mechanism and suggested a high importance in view of the efficient formation of secondary electrons during the interaction of ionizing radiation with biological matter. About 5 × 10^4^ electrons are produced per MeV deposited primary radiation in matter [3] and represent the most abundant of all secondary species produced. Their initial kinetic energy distribution should be also pointed out, with the vast majority generated in the energy range below 100 eV and peaking at near 9–10 eV [3]. In subsequent experiments, it turned out that the solvation state of the plasmid DNA is crucial for the strand break yield. The first experiments were carried out with lowest possible hydration level (i.e., almost dry DNA), and the mechanism of electron induced single strand breaks was recently confirmed by DEA experiments with a gas phase nucleotide analog [4]. However, electron irradiation experiments with hydrated oligonucleotides showed that electron induced strand breaks are strongly omitted in such environment [5]. Electrons, while still being quasi-free in solution, may induce bond cleavage in DNA constituents [6, 7], while electrons entering the prehydrated or hydrated stage turned out to be not effective in DEA to DNA nucleobases in solution [8].

The experiments by Sanche and co-workers initiated numerous experimental and theoretical studies with molecules of biological relevance [9]. Considering the DNA structure, possible targets for electrons represent the nucleobases, the sugar moiety and the phosphate group. Among the nucleobases, detailed electron attachment studies have been reported for the pyrimidine nucleobases thymine and uracil [10–13] as well as adenine [14, 15] and cytosine [16, 17]. Most of these studies, representing a sort of bottom-up approach, were carried out by means of crossed electron–molecule beam experiments with the biomolecules in the gas phase. They indicated as common properties that (i) the molecular anion is not observable on mass spectrometric timescales and (ii) the dehydrogenated parent anion is formed as most abundant product upon DEA. The latter anion is exclusively formed at low electron energies up to about 3 eV. In more details, for thymine, uracil and adenine a sharp peak structure near 1 eV was observed which was assigned to vibrational Feshbach resonances (VFR´s) with involvement of dipole-bound state [11]. In addition, a broader feature near 2 eV was also present, which was suggested to form by vibronic coupling of π* resonance with σ* states. This shape resonance mechanism dominates in cytosine [18]. In contrast, for guanine it was reported that the formation of CN^–^ is the predominant DEA reaction in free electron attachment to this nucleobase in the gas phase [15, 19, 20]. Among the other basic components of DNA, DEA studies were carried out with the sugar moieties in different forms like the ribose [21, 22], 2´-deoxyribose [23], and furan [24] as well as for the phosphate analogs like phosphoric acid esters [25]. In these studies, it was shown that also these DNA components may be strongly decomposed by low-energy electrons with kinetic energies below 15 eV.

Following the bottom-up approach, several DEA studies with the combined nucleobase and sugar moiety, i.e., the nucleosides, were carried out in the past. The focus was laid on the nucleoside based on the nucleobases thymine and uracil, i.e., thymidine (Td) [26–29] and uridine [30, 31], respectively. In the case of Td, following an early work by Illenberger and co-workers [26], Ptasinska et al. reported three anionic species formed upon DEA, (i) the dehydrogenated parent anion (Td-H)^−^ at m/z 241, (ii) the negatively charged thymine moiety at m/z 125 and (iii) the negatively charged deoxyribose moiety with two additionally abstracted hydrogen atoms at m/z 115 [27]. While the anions at m/z 241 and 115 were found exclusively at electron energies near about 1 eV, the negatively charged thymine moiety at m/z 125 was observed by two broad overlapping peaks in the electron energy from about 5–10 eV. Important to note is that Ptasinska et al. suggested the presence of certain peaks in the reported anion efficiency curves as a result from electron attachment to thermally decomposed thymidine [27]. In that study, like in most other electron attachment investigations with neutral biomolecules in the gas phase, the solid sample was resistively heated in an oven placed in vacuum. In particular, the anion efficiency curve of the negatively charged thymine moiety at m/z 125 indicated the thermal lability of Td due to the presence of the peak structure below 3 eV. This peak structure was identical to that in DEA to the nucleobase thymine. The relative intensity of this ion yield was also strongly dependent on the heating temperature of the oven which supported the suggestion of a thermal decomposition process [27]. Indeed, a later study by Bald et al. using laser induced acoustic desorption to gently transfer thymidine molecule into the gas phase showed that negatively charged thymine moiety at m/z 125 is predominantly formed at higher electron energies [28]. This anionic species was the only one observed within the detection limit of their experiment.

**Fig. 1:**
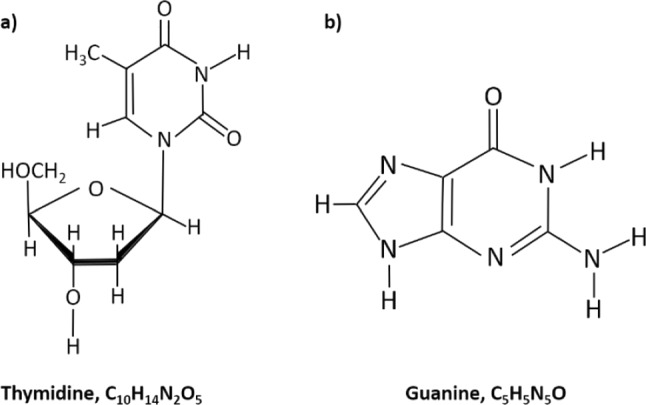
Molecular structures of (**a**) the nucleoside thymidine (Td) and (**b**) the nucleobase guanine (G)

In the present study, we revisited electron attachment to Td (see Fig. 1 for the molecular structure) using a newly developed laser desorption source (LDS) coupled to time-of-flight mass spectrometry. Instead of using a pulsed laser for the irradiation of sample deposited on a thin metal foil like in [28], we employed a continuous laser beam in the present experiment. Using an electron source with variable electron energy, we report the formation of 10 fragment anions upon DEA to Td. We also compare the LDS results with the anion yields from DEA to Td sublimed in a standard molecular beam oven (MBO) using the same experimental setup. When using the LDS, the negatively charged thymine moiety at m/z 125 is predominantly formed at higher electron energies above 5 eV. This result supports previous conclusions that abundant resonance features at lower electron energies may form upon DEA to thermal decomposition products.

In addition to Td, we also present the results from our electron attachment studies with laser-desorbed guanine (see Fig. 1 for the molecular structure). Previously, guanine was scarcely studied in the terms of electron attachment compared to other nucleobases. Within the detection limit of the experiment, we were able to observe four fragment anions. The results indicate that like for all other nucleobases, the dehydrogenated parent anion represents the most abundant anionic species upon electron attachment.

## Experimental methods

The experiments were carried out using a newly developed crossed electron–molecular beam setup. It consists of a neutral molecular beam source based on laser desorption, a Nier type ion source, a time-of-flight mass analyzer (TOF), and a multi-channel plate detector (MCP). The LDS for transferring neutral molecules into the gas phase is based on a principle design used previously in photoionization experiments [32]. Thymidine and guanine samples were purchased from Merck (Vienna, Austria) which stated purities of 99% and 98%, respectively. Both samples were used as delivered, mixed with H_2_O and deposited on thin stainless steel foil (thickness 12.5 µm) and dried at room temperature. The sample thickness was about 20–50 μm. Once dried, the foil was mounted onto a foil holder and placed into the ion source. For laser desorption a 445.7 nm cw laser diode from KWANT was used. The laser diode has a maximum output power of 1650 mW. The laser light was entering the vacuum chamber through a borosilicate glass window and was striking the backside of the stainless steel foil, coated with the sample on the other side. The laser irradiation process causes emission of a molecular plume in direction of the interaction region with the electron beam. For the current experiments a maximum desorption laser intensity of 3.5 W/cm^2^ was used. Previous multi-photon ionization experiments with adenine observed signatures of thermal decomposition only at significantly higher desorption laser intensities (>~6 W/cm^2^) [33]. Decomposition at high powers may be possible since the laser desorption by a continuous laser beam represents a local thermal heating process of the sample instead of the mechanical shake-off process as suggested for laser induced acoustic desorption [33]. Complementary measurements for Td were also performed using a MBO with resistively heated sample container. In these experiments the Td sample was heated to 414 K. The experiments were conducted under ultra-high vacuum conditions, with a base pressure as low as 10^−8^ mbar.

The ion source used in the course of the present experiments is a conventional Nier type ion source providing maximum electron currents up to 1 mA. Typical electron currents used for the study of negative ions were about 10–20 µA (achieved at a few eV) measured by a Faraday cup placed after the interaction region with the electron beam and connected to a picoammeter. The ions formed were locally extracted from the interaction region and transferred by an ion optics composed of several triple lens elements to the extraction region of the commercial TOF from Kore Technologies. Anions were orthogonally extracted with a frequency of 8 kHz into the TOF-region of the reflectron and finally detected by the MCP in chevron configuration.

The presented anion efficiency curves were obtained by collecting the mass spectra while scanning the electron energy. The energy scale was determined by measuring the ion yield of a calibration gas under identical conditions. The following electron attachment reactions were used for this calibration method [34]:

1 $$ {\text{SF}}_{{6}} + {\text{ e}}^{-} \to {\text{ SF}}_{{6}}^{-} $$

and

2 $$ {\text{SF}}_{{6}} + {\text{ e}}^{-} \to {\text{ F}}^{-} + {\text{ SF}}_{{5}} $$

The ion yield of SF_6_^−^ exhibits a sharp peak at about 0 eV originating from s-wave attachment to the neutral molecule, while F^−^ formation occurs via three resonance states in the electron energy range between 5.5 and 11.5 eV [34]. The electron energy resolution was determined as the full-width at half-maximum of the SF_6_^–^ peak (about 1 eV). We estimate the accuracy of the reported peak positions to be ± 0.5 eV.

## Results and discussion

### Thymidine

**Fig. 2:**
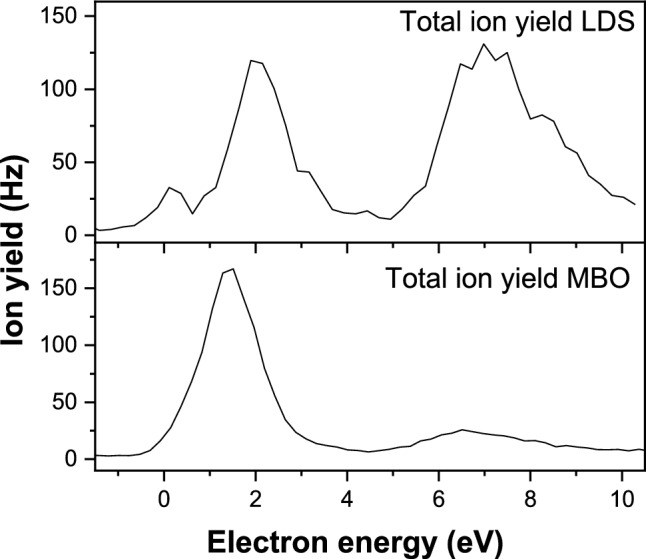
Total ion yield as function of the initial electron energy upon electron attachment to thymidine vaporized upon laser desorption source (LDS), upper panel, and resistively heated molecular beam oven (MBO), lower panel

Using the mass spectrometric setup described above, we studied electron attachment to thymidine (Td) in the electron energy range of about 0–10 eV. Figure 2 shows the total ion yield of anions formed upon electron attachment to Td, for two different methods to generate Td targets in the gas phase, (i) LDS, upper panel, and (ii) sublimation in the resistively heated MBO, lower panel. Such spectra for the total ion yield indicate the range of electron energies, at which anions from the molecule are formed, i.e., they also provide information of the temporary negative ion states of Td. The spectra indicate that negative ions are mainly formed in two regions, near 1–2 eV and about 6–10 eV. The LDS data also show a minor feature near zero eV, which could be obscured in the MBO data due to the high abundance of the main peak at slightly higher electron energy. Formation of anions at the higher electron energy range 6–10 eV is likely accompanied by (electronic) core excitation, i.e., after attachment of the excess electron also an electron of the molecule is located in a formerly unoccupied orbital. The direct comparison of the data for the different sources in Fig. 2 clearly indicates that the relative abundance of the signal at low electron energies and high electron energies strongly depends on the neutral source. Previous studies reported that Td is a thermally labile molecule and decomposes at elevated heating temperatures [27, 35]. Based on photoionization studies, Hevola et al. suggested that the onset of thermal decomposition can be found at 411 K [35]. Ptasinska et al. studied the thermal effect in more details for electron attachment to Td and proposed that the glycosidic bond rupture between the base and sugar moiety occurs with subsequent hydrogen transfer to generate closed shell thymine molecules [27]. The latter would efficiently capture electrons with low energies between 1 and 2 eV. The present MBO data were recorded at the oven temperature of about 414 K and thus a significant fraction of Td molecules will be decomposed before interacting with the electron beam. Considering now the LDS spectrum, the abundance of low- and high-energy features is nearly equal. Thus, the laser desorption is a much milder way to transfer the Td molecules into the gas phase than the heating in the MBO at 414 K. The total ion yield spectrum in the upper panel of Fig. 2 may be also compared with the total (dissociative) electron attachment cross sections for molecular constituents of Td reported by Aflatooni et al. [17]. Similar to the present data, the total cross section shape for thymine showed features at low energy near 1−2 eV and high energy above 5 eV. As sugar analog Aflatooni et al. studied tetrahydrofuran (THF), which turned to have a much lower cross section than thymine, as well as, closer to the sugar moiety of Td due to addition of OH group, 3-hydroxyTFH. For the latter molecule, the total cross section was on the same order of magnitude like that of thymine, but just showing high energy features. For thymine, the cross section below about 3 eV resembled closely the peak structure obtained in the anion efficiency curve of the dehydrogenated parent anion, composed of a rather narrow peak near 1 eV, overlapping with a broad peak at 1.7 eV [17]. Assuming a simple summation rule for the total cross section to predict the Td total cross section shape from the single constituents, low and high energy features should be on the same order of magnitude, which is also the case in our LDS data.

**Fig. 3:**
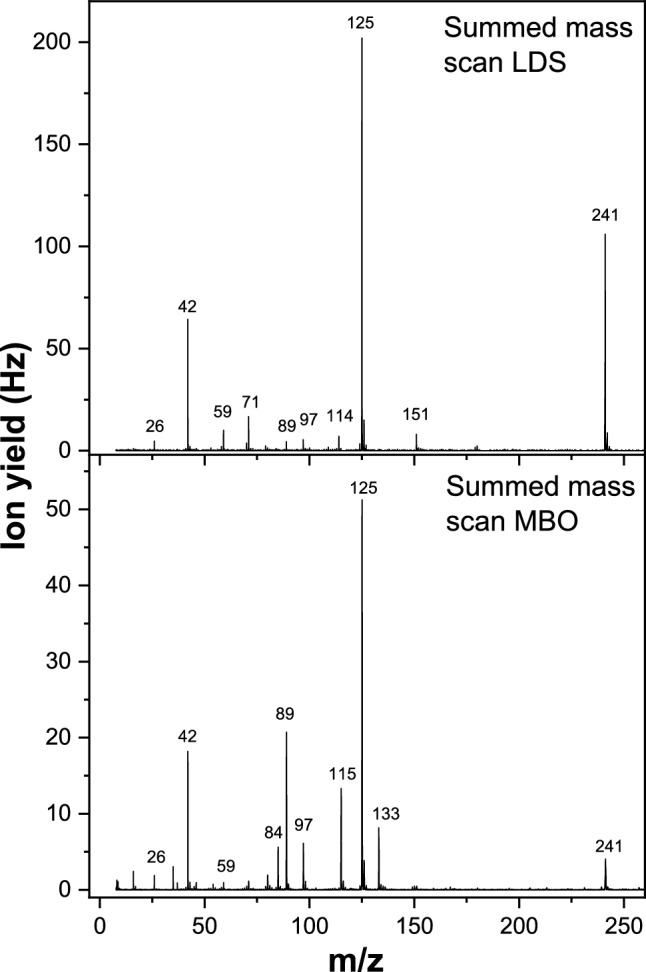
Summed mass scan, measured in the electron energy range from about zero to 10 eV in steps of 0.3 eV, for electron attachment to thymidine sublimed in laser desorption source (LDS), upper panel, and resistively heated molecular beam oven (MBO), lower panel. Relevant peaks are labeled by their m/z ratio

To shed more light on the question, which fragment anions are formed from intact Td and which ones from (to a fraction) decomposed Td, summation of mass scans for LDS and MBO are plotted in Fig. 3. Since electron attachment is a resonance process, the summed mass spectra provide an overview, which anions are formed for the different neutral sources. No parent anion of Td is observable irrespective of the neutral source. Just a slightly positive adiabatic electron affinity of about 0.4 eV was reported for Td [36, 37], resulting in a bound valence state of the Td anion observed in photoelectron spectroscopy measurements [38]. However, another study predicted a vertical attachment energy of 0.11 eV for the π_1_* state of Td [29]. The missing parent anion signal in our experiments indicates that the Td transient negative ion (TNI) formed upon free electron attachment is insufficiently stable against spontaneous electron detachment. The direct comparison of the summed mass spectra in Fig. 3 indicates that the major peaks observed in the LDS spectrum, i.e., m/z 241, 125 and 42, are also present in the MBO spectrum. These m/z can be assigned to the dehydrogenated parent anion (Td–H)^−^, the thymine moiety (T–H)^−^, and OCN^−^, respectively. However, Fig. 3 indicates that the ratio of the corresponding fragment anion intensities is dependent on the neutral source. In particular, the LDS spectrum shows that the intensity of (Td–H)^−^ at m/z 241 is strongly enhanced relative to the other peaks in the LDS spectrum. In addition, there are also mass peaks, which are present with considerable intensity in just one of the spectra. This is the case for peaks at m/z 114 and 151 in the LDS spectrum, while peaks at m/z 84, 115 and 133 are apparent just in the MBO spectrum. The peak at m/z 125 assigned to the formation of the thymine moiety (T–H)^−^ is the most abundant in both spectra shown in Fig. 3. The thymine ring may also decay, which would explain the presence of some less abundant peaks labeled in the upper panel of Fig. 3. The assignments m/z 26 to CN^−^, m/z 97 to C_4_H_5_N_2_O^−^ as well as the major peak at m/z 42 to OCN^−^ in the LDS spectrum seem straightforward since those species were also suggested to be resultant from the DEA to the single thymine molecule [39]. Just for m/z 71 another structure, C_2_HNO_2_^−^, compared to Ref. [39] needs to be suggested since the previously proposed C_2_H_3_N_2_O^−^ is not possible for Td due to missing H atom at N1 nitrogen position. In addition, we assign two peaks in the LDS mass spectrum to fragments of the sugar moiety, m/z 59 (C_2_H_3_O_2_^−^) and m/z 89 (C_3_H_5_O_3_^−^), which are formed by ring cleavage. For the last two species labeled in the LDS mass spectrum shown in Fig. 3, m/z 114 and m/z 151, we suggest intact glycosidic bond. In the case of m/z 114, possible structures would be C_3_H_2_N_2_O_3_^−^ and/or C_4_H_4_NO_3_^−^ which are formed by the fragmentation of the sugar as well as the nucleobase moieties. The peak at m/z 151 could form from the intact thymine moiety with C_2_H_2_ fragment from the sugar, i.e., C_7_H_7_N_2_O_2_^–^.

**Fig. 4:**
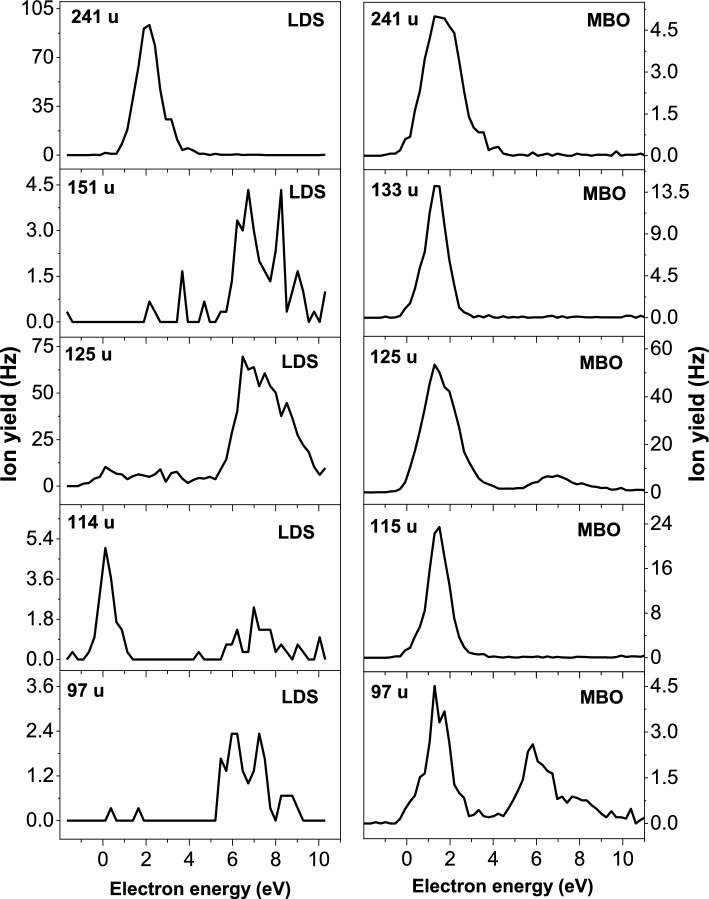
Anion efficiency curves for fragment anions with masses 97–241 u formed upon electron attachment to thymidine vaporized upon laser desorption source (LDS), left panels, and resistively heated molecular beam oven (MBO), right panels

**Fig. 5:**
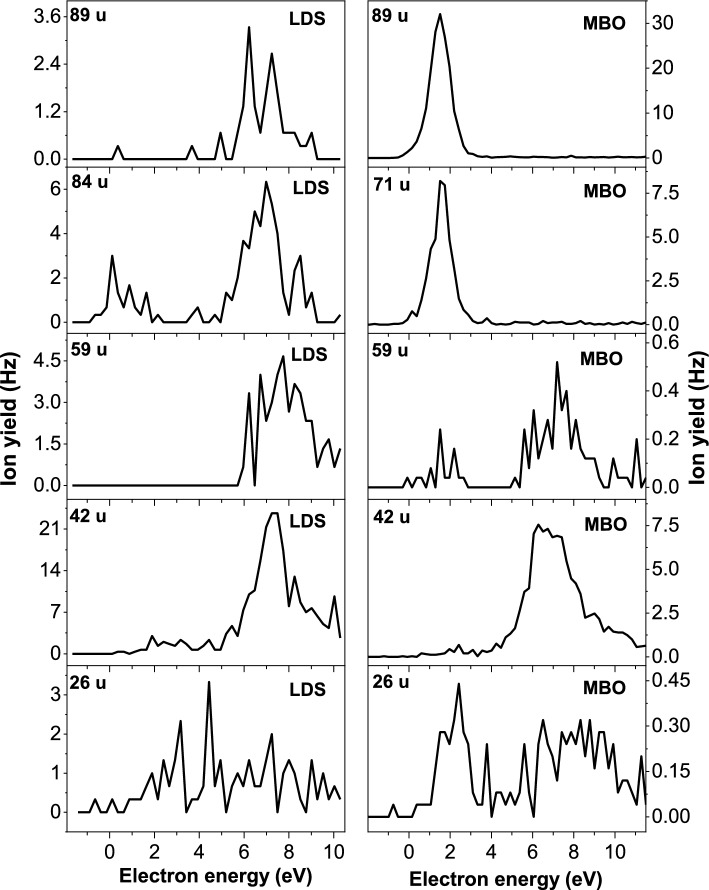
Anion efficiency curves for fragment anions with masses 26–89 u formed upon electron attachment to thymidine vaporized upon laser desorption source (LDS), left panels, and resistively heated molecular beam oven (MBO), right panels

Figures 4 and 5 show the corresponding anion efficiency curves of the most abundant anions detected in the mass spectra. The anion efficiency curve for each fragment anion is compared for the LDS and MBO sources, if formed with considerable amount. As mentioned in the discussion of total ion yield, the fragment anions are mainly formed in two regions, near 2 eV and about 6–10 eV. In the LDS efficiency curves, the dehydrogenated parent anion (Td–H)^−^ appears with the highest intensity of all fragment anions, although in summed mass spectrum shown in upper panel of Fig. 3 it is just second abundant one, after (T–H)^−^. This difference can be explained by broader width of the high energy feature in the (T–H)^−^ anion efficiency curve, making it to the most abundant in the summed LDS spectra. The position of the main peak in the (Td–H)^−^ anion efficiency curve seems independent from the neutral source, indicating that thermal excitation lowering the thermodynamic threshold does not play a significant role in the dissociation process. The most dramatic change in the peak intensities at low and high electron energies is observed for the thymine moiety (T–H)^−^ (see Fig. 4) which is a product from electron attachment to Td mainly at high energies, while it is formed from the thermal decomposition product at low energies. Another notable change is also found for C_4_H_5_N_2_O^−^ (97 u) in Fig. 4, which is predominantly formed at high electron energies in the LDS data. In contrast, the MBO data reveal an abundant peak near 1–2 eV and high energy features near 6 and 8 eV. While the two peaks at high energy can be assigned to the intact thymine nucleobase [39], which is formed as decomposition product, the low-energy peak may indicate the presence of other thymine based decomposition products in the beam.

Figure 5 shows the anion efficiency curve of fragment anions with lower mass. For the anion with the mass 89 u (C_3_H_5_O_3_^−^) the same effect in terms of preferred resonance energy can be observed like for (T–H)^−^ (mass 125 u), though for C_3_H_5_O_3_^–^ we suggest localization of excess charge at the sugar moiety. The lightest fragment anions, C_2_H_3_O_2_^–^ (mass 59 u), OCN^−^ (mass 42 u) and CN^−^ (mass 26 u), reveal on the other hand similar anion efficiency curves for LDS and MBO. The latter two anions are formed in a broad electron energy range between about 2 and 10 eV. In the case of thymine, it was previously suggested that OCN^–^ forms upon a sequential decay with initial loss of H from nitrogen sites in the transient negative ion [40]. The N3 position would be still available in Td for such sequential decay. The ratio of (Td–H)^–^ and OCN^–^ in the LDS spectra is ~5 (peak maxima). The ratio of the dehydrogenated parent anion and OCN^–^ is very similar for the single nucleobase thymine [39], i.e., the efficiency of such sequential decay involving also cleavage of the pyrimidine ring would be conserved at the step from nucleobase to nucleoside.

Finally we note that a detailed study presenting the anion efficiency curves of Td heated to 411 K was carried out by Muftakhov et al*.* [29]. In their work, they pointed out that humidity of the sample has an effect on thermal decomposition of Td and thus they performed extensive drying in vacuo, by heating to lower, pre-evaporation temperatures [29]. Indeed, the present LDS data resemble their reported anion efficiency curves more than the MBO data and overall agreement is observed.

### Guanine

**Fig. 6:**
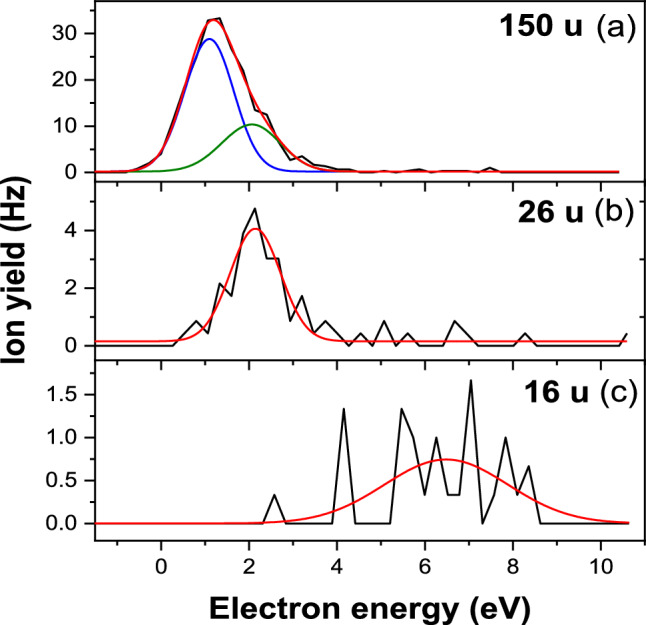
Efficiency curves of the anions formed upon electron attachment to laser-desorbed guanine, **a** (G-H)^−^ (15 u), **b** CN^−^ (26 u), and **c** O^−^/NH_2_^−^ (16 u). Black line experimental data; colored lines- peak fits

Figure 6 shows the resulting anion efficiency curves measured for electron attachment to laser-desorbed guanine (G) sample in the electron energy range from about zero to 10 eV. Within the detection limit of the experiment, we were able to detect four anions. While for (G-H)^−^ and CN^−^ the resonance structure is well discernible, the measurement statistics for O^−^/NH_2_^−^ is rather limited. For OCN^–^ the situation is even worse due the low signals observed and thus we show the corresponding anion efficiency curve only as supplementary material (Fig. S1). In addition, we discuss the anion efficiency curve of OCN^−^ only qualitatively, while for the other fragment anion we report the peak position(s) obtained by Gaussian fits in Table 1.

**Table 1:** List of the peak positions for anions observed from electron attachment to G, together with the mass

Mass (u)	Anion species	Peak position (eV)	
150	(G-H)^−^	1.1	2.1
26	CN^−^	2.1	
16	O^−^/NH_2_^−^	6.5	

In the course of the present experiments we did not find any anion signal for the parent anion of G. This may result from the experimental detection limit, however it was reported that the (valence) adiabatic electron affinity of G is negative [41] and thus parent anion signal is not expectable on mass spectrometric timescales. However, predictions exist that G should form a dipole-bound anion upon electron attachment [41, 42]. The most stable tautomer of G has the canonical form with a large dipole moment of 6.3 D and an electron affinity of 85 meV [43]. Compared to valence-bound anions, the binding energy of dipole-bound states is very low and thus the dipole-bound anion cannot be detected in the present experiment due to earlier autodetachment. However, a dipole-bound state may act as doorway state to DEA as proposed by Sommerfeld for uracil [44]. Based on experimental data, Burrow et al*.* applied the model of this doorway mechanism to explain a narrow peak structure near 1 eV obtained in the anion efficiency curve of the dehydrogenated parent anion of thymine and uracil [11]. This doorway mechanism may also be effective for G since we observe the dehydrogenation as the most abundant DEA reaction in electron attachment to G. The resulting anion efficiency curve of (G-H)^−^ shown in Fig. 6a reveals a major peak located at 1.1 eV. The peak is slightly asymmetric which may indicate another weaker resonance feature. The fitting procedure based on (Bi-) Gaussian fit functions suggests a second peak at 2.1 eV. At this energy, in contrast to the dipole-bound doorway state at about 1 eV, coupling of a valence π* state with the dissociative state may occur. Such situation was also suggested for the other purine compounds adenine and hypoxanthine, since the peak structure composed of narrow peaks (originating from dipole-bound state) and wide peak (from π* state) additionally supported these assignments [14, 45]. Due the limited energy resolution of the presently used ion source the peak widths cannot be considered for a conclusion, if the same mechanisms are operative. Recent electron scattering calculations predicted the three lowest π* states at 0.97 eV, 1.47 eV, 2.65 eV [46], in good agreement with a previous theoretical study predicting 0.9 eV, 1.55 eV and 2.75 eV [47]. The deviation of the present feature in the anion efficiency curve at 2.1 eV and the predicted π_2_* energy of 1.55 eV may be considered as large but it should be noted that two different quantities are compared and for example the dissociation process has autodetachment of the excess electron as competition, which depends on the initial electron energy [34]. Thus, DEA peaks may be shifted compared to the initial resonance energies.

For dehydrogenation, several sites in the guanine molecule need to be considered. Few theoretical studies on the calculated thresholds of (G-H)^−^ for different positions of H abstraction are available [20, 46, 48]. According to the most recent study [46], three different nitrogen sites would be possible for the peak at about 1 eV since the reported threshold energies are 0.83 eV, 0.94 eV and 0.97 eV for hydrogen abstraction from N9, N1 and amino group position, respectively (zero-point energy corrected value at 0 K). Considering the dynamics involving a dipole-bound doorway state mentioned above, we propose involvement of amino group in the formation (G-H)^−^ at about 1 eV, since the diffuse orbital of the dipole-bound anion is pointing toward this group in the canonical G [43].

As pointed out in the introduction, experimental electron attachment studies with guanine are scarce. Minaev et al*.* reported the total cross section for electron attachment up to the electron energy of about 5 eV [20]. Their measurement with an electron energy distribution of 0.3 eV indicated a narrow peak at about 1 eV followed by a broad feature at about 2.3 eV with about 40% maximum intensity of the main peak. This result would agree with the present observation, however it should be noted the relative abundance of the narrow VFR´s will be underestimated for a worse energy width of the electron beam [49]. The only previous study reporting the anion efficiency curves of mass selected fragment anions was carried out by Abdoul-Carime et al*.* who compared the DEA properties of the purine nucleobases adenine and guanine [15, 19]. The beam of guanine molecules emanated from an oven heated to 500 K. They reported anion signals for formation of CN^−^, OCN^−^, (G-O/NH_2_)^−^, (G-H)^−^, O^−^/NH_2_^−^ and (G-HOCN)^−^ (listed by intensity of the most abundant peak in the anion efficiency curve). Except O^−^/NH_2_^−^, all anions were formed predominantly at electron energies below about three eV. It was pointed out in [15] that for adenine the dehydrogenation channel represents about 95% of the total yield while in striking contrast the dehydrogenation reaction represents only about 5% of the total yield for guanine. The observation of the high abundance of the dehydrogenation reaction in the case of adenine was also supported later by a detailed DEA study using two different experimental setups [50]. A close view of the (G-H)^−^ ion yield measured by Abdoul-Carime et al*.* is shown in Ref. [19] and reveals the main peak at about 1.7 eV. From the asymmetry of the peak with a weak tail at lower energy the authors concluded that there is another feature at 0.9 eV. Thus, the contribution from the dipole-bound state seems to strongly suppressed in the previous measurement of (G-H)^−^. We ascribe this effect to the high evaporation temperature used in the previous studies (450–500 K) which leads to the presence of other tautomers in the molecular beam. As predicted by Tripathi and Dutta [43], those tautomers have a lower dipole moment and lower electron affinity, and thus the doorway mechanism may be less effective in this case.

In the present electron attachment study with laser-desorbed G, we observe CN^−^ as the second most abundant fragment anion. The corresponding anion efficiency curve indicates a single peak located at about 2.1 eV, see Fig. 6b and Table 1. The relative intensity of the peak maximum is about 15% of the maximum of (G-H)^−^. In contrast to the dehydrogenation representing a single bond cleavage, the formation of CN^−^ requires excision from one of the two rings which may be also accompanied by formation of new molecular bonds. The reaction is also driven by the tremendous electron affinity of the pseudohalogen CN (about 3.86 eV [51]), which makes it possible to form CN^−^ at about 2 eV. In lack of detailed quantum chemical calculations for G, we may compare with the previous results for the guanine derivate 8-oxo-guanine [46]. For this compound, the threshold of CN^−^ formation upon DEA was predicted to be 0.87 eV for excision from the imidazole moiety, indicating that the CN^−^ formation may indeed feasible at low electron energies. We just note that the experimental anion efficiency curve of CN^−^ from 8-oxo-guanine also showed just one single peak near 2 eV which was ascribed to the initial formation of the π*_2_ resonance [46]. In contrast, CN^−^ was observable in DEA to hypoxanthine just above about 4 eV (main peak at 6.2 eV), like several other fragment anions of this molecule [45]. In the present results we find the formation of ion signal at m/z 16 upon a single peak at 6.5 eV, see Fig. 6c. The question, if O^−^, NH_2_^−^ or both fragment anions be formed was addressed in the previous study by Abdoul Carime et al. [15]. Taking the well-known electron affinities of oxygen atom and amino group and the bond dissociation energies of C=O and C–NH_2_ from literature, they estimated the thresholds 6.1 eV and 3.1 eV for formation of O^−^ and NH_2_^−^, respectively. Similar to the present data, they observed a peak at 7.0 eV and concluded that both anions may form upon a core-excited resonance. In addition, they observed a weaker peak at 2.0 eV, which is not observed here within the detection limit.

Finally, we note that we also obtained very low anionic signal at m/z 42, which can be assigned to OCN^−^. Like CN, OCN represents a pseudo halogen which means that the radical has a high electron affinity of about 3.6 eV [51]. However, while for CN eight sites of formation in G are available from structural point of view, OCN can form in G at just one specific site by break-up of the pyrimidine moiety. This may lead to the presently observed much lower anion yield of OCN^−^ compared to CN^−^, see Figures S1 and 6b, respectively. The same tendency was also observed for hypoxanthine, though for this compound the anion efficiency curves of OCN^−^ and CN^−^ exclusively showed core-excited resonances [45]. For G, the above mentioned π_2_* resonance at low electron energies near about 2 eV seems to be a precursor state for the complex decomposition of G into both fragment anions.

## Conclusions

In the present study on free electron attachment to thymidine and guanine we employed the laser desorption technique in order to transfer the biomolecules into the gas phase. The results for Td indicate that previously obtained thermal decomposition can be avoided by the use of laser desorption with a continuous laser beam. Thus, previous conclusions from multi-photon ionization experiments that Td cannot be desorbed without thermal decomposition [52] are not supported by the present results. The use of a continuous laser beam seems favorable to raise the desorption yield as long as no excessive laser powers are used which thermally degrade the sample. Previous photoionization studies with the nucleobase adenine indicated a doubling of the parent cation signal when a cw laser instead of a ns laser is used [33].

With respect to thermal degradation, G was suggested to be a labile compound, for example Aflatooni et al. mentioned that in their study of total attachment cross sections the attempts with G were unsuccessful, with considerable indication that the sample partially decomposed [17]. Moreover, they pointed out in Ref [17] that the previously reported anion efficiency curves for DEA to G [15] could have been affected by thermal decomposition of G since the (G-H)^–^ yield was an order of magnitude smaller than the OCN^−^ yield. In the present result for laser-desorbed G we obtain a reversed ratio of (G-H)^–^ and smaller fragment anions like OCN^−^. We would like to point out an alternative explanation for the different ratios obtained, which involves the presence of different tautomers in the experiments. The energetically most stable tautomer has a high dipole moment, and the resulting dipole-bound anion may serve as a doorway state for the dehydrogenation process. This may lead to the high abundance of (G-H)^–^ in the present experiment. It was also noted in electron transmission studies with resistively heated G [53], that the results did not match the molecular orbital calculations and suggested the formation of other tautomers in the gas phase.

## Supplementary Information

Below is the link to the electronic supplementary material.

Supplementary file1 (XLSX 967 KB)

Supplementary file2 (PDF 123 KB)

## Data Availability

The data that support the findings of this study are available within the article and its supplementary material. This manuscript has associated data in a data repository (10.5281/zenodo.15605899).
