# Supplementary material for: Electron interaction with laser-desorbed thymidine and guanine in the gas phase
Source: Eur Phys J D At Mol Opt Phys. 2025 Jun 30;79(6):76. doi: 10.1140/epjd/s10053-025-01023-9 (PMC12208974; doi:10.1140/epjd/s10053-025-01023-9)
Supplement: Supplementary file 2 — Supplementary file2 (PDF 123 KB) [file 10053_2025_1023_MOESM2_ESM.pdf]

Supplementary Material

for

Electron interaction with laser desorbed thymidine and guanine in the gas phase

Debasish Parida[1,2], Jiakuan Chen,[1,2] Lara Schorr,[1,2] Vy T.T. Nguyen,[1,2] Muhammad Saqib,[1,2] Andreas Bayer,[1,2] Fabio Zappa[1] and Stephan Denifl[1,2*]

[1]Institut für Ionenphysik und Angewandte Physik, Universität Innsbruck, Technikerstrasse 25, A-6020 Innsbruck, Austria

[2]Center for Molecular Biosciences Innsbruck, Universität Innsbruck, Technikerstrasse 25, A-6020 Innsbruck, Austria

*corresponding author: Stephan.Denifl@uibk.ac.at

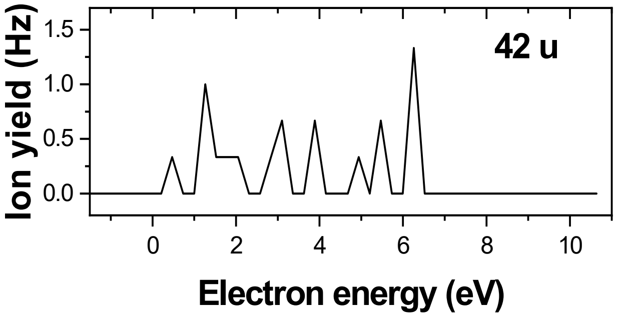

Figure S1. Efficiency curve of the OCN^- anion (42 u) formed upon electron attachment to guanine. Black line-experimental data.
